# Long COVID (post-COVID-19 condition) in children: a modified Delphi process

**DOI:** 10.1136/archdischild-2021-323624

**Published:** 2022-04-01

**Authors:** Terence Stephenson, Benjamin Allin, Manjula D Nugawela, Natalia Rojas, Emma Dalrymple, Snehal Pinto Pereira, Manas Soni, Marian Knight, Emily Y Cheung, Isobel Heyman, CLoCk Consortium, Roz Shafran

**Affiliations:** 1 UCL Great Ormond Street Institute of Child Health Population, Policy and Practice, London, UK; 2 National Perinatal Epidemiology Unit, Oxford, UK; 3 MRC Unit for Lifelong Health and Ageing at UCL, UCL Great Ormond Street Institute of Child Health Library, London, UK; 4 Research Department of Medical Education, UCL Medical School, London, UK

**Keywords:** COVID-19, adolescent health, child health

## Abstract

**Objective:**

The aim of this study was to derive a research definition for ‘Long COVID (post-COVID-19 condition)’ in children and young people (CYP) to allow comparisons between research studies.

**Design:**

A three-phase online Delphi process was used, followed by a consensus meeting. Participants were presented with 49 statements in each phase and scored them from 1 to 9 based on how important they were for inclusion in the research definition of Long COVID in CYP. The consensus meeting was held to achieve representation across the stakeholder groups. Statements agreed at the consensus meeting were reviewed by participants in the Patient and Public Involvement (PPI) Research Advisory Group.

**Setting:**

The study was conducted remotely using online surveys and a virtual consensus meeting.

**Participants:**

120 people with relevant expertise were divided into three panels according to their area of expertise: Service Delivery, Research (or combination of research and service delivery) and Lived Experience. The PPI Research Advisory group consisted of CYP aged 11–17 years.

**Main outcome measures:**

Consensus was defined using existing guidelines. If consensus was achieved in two or more panels or was on the border between one and two panels, those statements were discussed and voted on at the consensus meeting.

**Results:**

Ten statements were taken forward for discussion in the consensus meeting and five statements met threshold to be included in the research definition of Long COVID among CYP. The research definition, aligned to the clinical case definition of the WHO, is proposed as follows: *Post-COVID-19 condition occurs in young people with a history of confirmed SARS-CoV-2 infection, with at least one persisting physical symptom for a minimum duration of 12 weeks after initial testing that cannot be explained by an alternative diagnosis. The symptoms have an impact on everyday functioning, may continue or develop after COVID infection, and may fluctuate or relapse over time*. The positive COVID-19 test referred to in this definition can be a lateral flow antigen test, a PCR test or an antibody test.

**Conclusions:**

This is the first research definition of Long COVID (post-COVID-19 condition) in CYP and complements the clinical case definition in adults proposed by the WHO.

## Abstract (precis)

How might one understand long covid in children? One way is to arrive at a consensus on a research definition. The authors describe a modified Delphi approach, which included people with lived experience, research and service delivery.

**What is already known on this topic?**

- Definitions for Long COVID vary in number, type and duration of symptoms.
- Prevalence estimates for Long COVID are inconsistent, ranging from 1% to 51%.
- A consistently applied definition of Long COVID will help reduce the variability of prevalence estimates.

**What this study adds?**

- The first research definition of Long COVID in children and young people that complements the definition proposed by the WHO for adults.

**How this study might affect research, practice or policy?**

- Ability to reliably compare and evaluate studies on prevalence, course and outcome of Long COVID in CYP using this definition.

## Introduction

There is growing recognition that the COVID-19 pandemic has left a significant proportion of the population experiencing symptoms in the long term. Such symptoms are termed post-COVID-19 condition or ‘Long COVID’ with the former terminology being considered least controversial and preferred by the WHO (the term Long COVID is used throughout the manuscript as this was the term used in Delphi consensus process. The term is considered synonymous with post-COVID-19 condition.). Acute SARS-CoV-2 infection in children and young people (CYP) is usually asymptomatic^1^ or mild^2^ compared with adults.^3^ More children^2^ recover without sequelae compared with adults.^3^ Over 200 symptoms have been associated with long COVID^4 5^ in adults but the most common symptoms in both adults and children are similar, especially fatigue and headache. Estimates of the prevalence of Long COVID in CYP vary. A UK survey of self-reported Long COVID in 320 825 people reported a prevalence of 0.16% for 2–11 years, 0.65% for 12–16 years, and 1.22% for 17–24 years.^6^ A large national study of Long COVID in children, the CLoCk Study,^1^ found that at 3 months post-COVID-19 testing, 66.5% of CYP with a positive test and 53.3% of CYP with a negative test still had symptoms, at least one of which was physical, while 30.3% and 16.2%, respectively, had three or more symptoms.

It is currently unclear whether Long COVID represents one or many different conditions and it has consequently been difficult to derive a universally accepted definition for the condition.^7–14^ Definitions vary in the number and type of symptoms included, as well as the duration of symptoms.^2 9 15–17^ Research into the prevalence and impact of Long COVID has consequently been hampered, thereby delaying the implementation of policies and services that could help affected CYP.

The Delphi process is a well-established method for achieving consensus among groups of key stakeholders on questions relating to health sciences. It has been used to identify outcomes of importance for a range of conditions,^18–23^ define metrics for monitoring the quality of provision of care in the National Health Service (NHS),^24^ develop a UK-wide pathway for managing CYP with Paediatric Inflammatory Multi-system Syndrome Temporally associated with SARS-CoV-2 infection,^25^ and define conditions, including post-COVID-19 syndrome in adults.^26–28^ The WHO definition for post-COVID-19 condition in adults, derived using a Delphi process, is given in box 1.^29^ However, there are alternative definitions. The National Institute for Health and Care Excellence definition for adults is given in box 2.^30^

Box 1 **WHO clinical case definition of post-COVID-19 condition in adults**

People with a history of probable or confirmed SARS-CoV-2 infection, usually 3 months from the onset of COVID-19, with symptoms that last for at least 2 months and cannot be explained by alternative diagnoses.

Box 2 **National Institute for Health and Care Excellence clinical case definitions of post-COVID-19 syndrome and ‘Long COVID’ in adults**

Signs and symptoms that develop during or after an infection consistent with COVID‐19, continue for more than 12 weeks and are not explained by an alternative diagnosis. It usually presents with clusters of symptoms, often overlapping, which can fluctuate and change over time and can affect any system in the body. Post‐COVID‐19 syndrome may be considered before 12 weeks while the possibility of an alternative underlying disease is also being assessed.

In addition to the clinical case definitions, the term ‘Long COVID’ is commonly used to describe signs and symptoms that continue or develop after acute COVID‐19. It includes both ongoing symptomatic COVID‐19 (from 4 to 12 weeks) and post‐COVID‐19 syndrome (12 weeks or more).

Within the WHO definition, it was explicitly stated that a separate definition may be required for CYP. The justification for this is that less is known about Long COVID in young people. The emerging data indicate that there are similarities with the adult symptoms but also differences, for example, in the higher proportion of CYP who present without symptoms at the time of their initial infection.^1^ The aim of this study was therefore to use a three-phase online Delphi process followed by an online consensus meeting to derive a definition for Long COVID in CYP that could be used for research to allow comparisons between studies.

## Method

**Figure 1:**
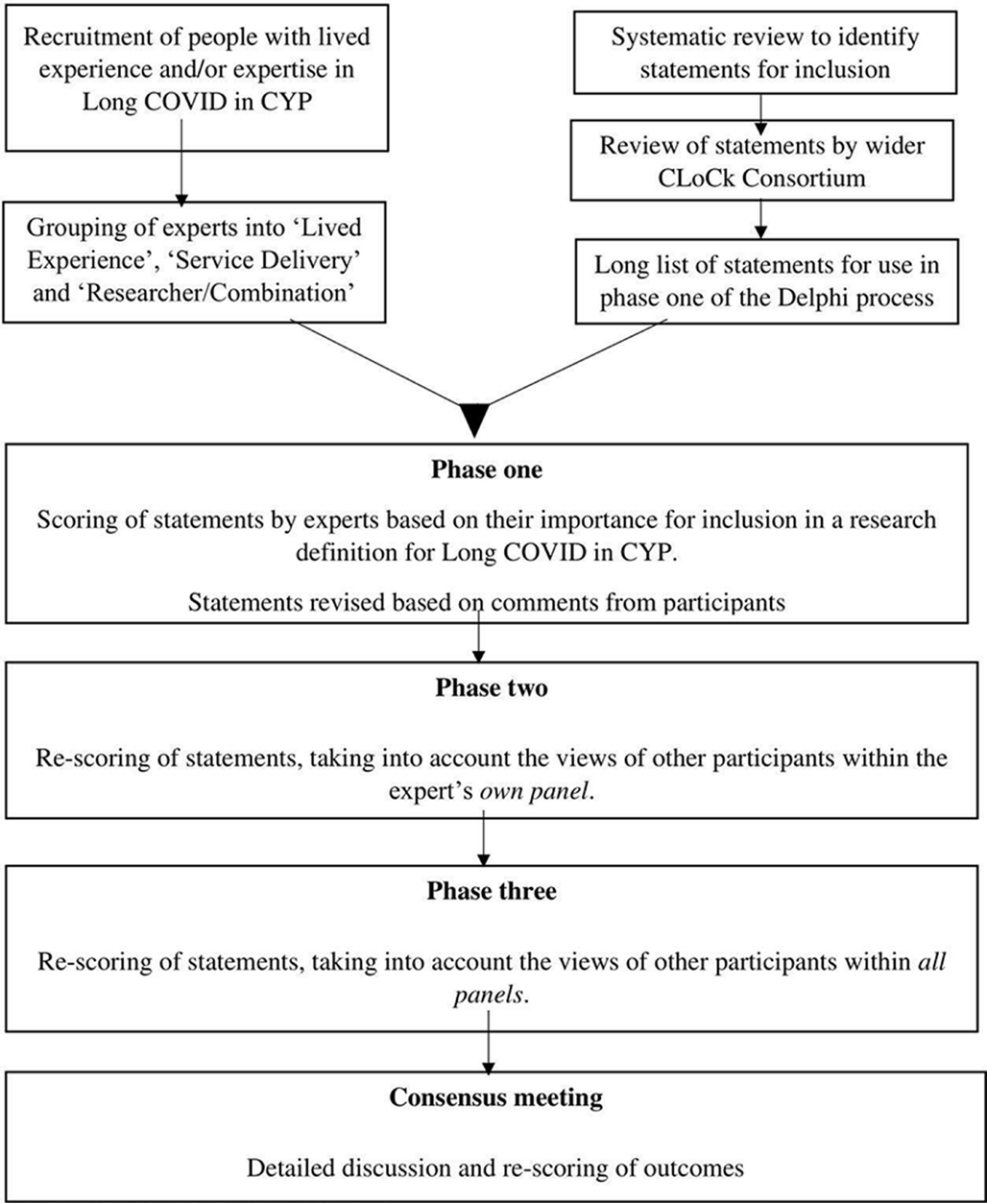
Consensus process. CYP, children and young people.

As per COMET,^31^ a three-phase online Delphi process followed by a consensus meeting, was conducted (figure 1). The scope of this consensus process was to develop a definition of Long COVID in CYP that could be used for research purposes. This definition was not intended to be used for the purposes of clinical referral, investigation or treatment.

### Participants

People with relevant expertise were identified through published materials, clinical organisations, support groups and professional bodies. A combination of direct invitations to participate and invitations via existing mailing lists were used. Those confirming their interest in participating were categorised into three panels according to their area of expertise: (1) Service Delivery, (2) Research (or a combination of research and service delivery) and (3) Lived Experience.

### Information sources

The survey consisted of 49 statements in eight categories, covering different areas of the definition of Long COVID in CYP. These statements were developed on the basis of existing literature, including an unpublished systematic review (May 2021–Lauren O’Mahoney, Leicester, personal communication), a NICE guideline on managing long effects of COVID-19,^11^ NHS advice on COVID^32^ and empirical data from the CLoCk Study.^1^

### Consensus process

An online three-phase Delphi process was conducted using Limesurvey^33^ and was followed by a virtual consensus meeting. In each phase, participants were presented with the 49 statements, accompanied by relevant empirical data from the CLoCk Study and existing literature, and were asked to score the statements from 1 to 9 based on how important the participants thought the statements were for inclusion in a research definition of Long COVID in CYP. Scores of 1–3 were classed as not important, 4–6 as important and 7–9 as very important. In phase one, participants were asked to score each statement based on their own opinion. In phase two, participants were shown graphical (ie, score histograms) and numerical (ie, the median score) representations of how others in their panel scored each statement and were given the opportunity to revise their score based on this information if they wished to do so. In phase three, participants were shown similar graphical and numerical representations of how all three panels had scored each statement and were again given the opportunity to revise their score if they chose to do so. Participants were given the opportunity to comment on the statements, and if they felt they did not have sufficient expertise to score a statement, could select ‘don’t know’ instead of assigning a score. Only those who fully completed a phase were invited to participate in the subsequent phase.

At the end of the third phase of the Delphi process, statements were defined according to the number of panels (ie, Service Delivery; Research (or a combination of research and service delivery) and Lived Experience) in which the threshold for ‘consensus important’ or ‘consensus unimportant’ had been reached. Consensus important was defined per COMET^31^ guidelines as ≥70% of participants scoring the statements 7–9 and <15% of participants scoring the statements 1–3. ‘Consensus unimportant’ was defined as ≥70% of participants scoring the statements 1–3 and <15% of participants scoring the statements 7–9. No statements were dropped or added between phases of the Delphi process.

### Consensus meeting

Participants who completed all three phases of the Delphi process were invited to the consensus meeting in a purposive manner to achieve spread across the stakeholder groups. The meeting was held virtually (Zoom Video Communications, V.5.1.0), and was independently chaired by an expert in consensus methodology. Statements that had achieved ‘consensus important’ status in two or more panels at the end of the Delphi process were automatically discussed and voted upon for inclusion in the definition of Long COVID in CYP. Other statements could be promoted for discussion and scoring by the attendees as long as they had not met the threshold for ‘consensus unimportant’ status in two or more panels at the end of the Delphi process.

Those statements that 70% or more of the consensus meeting participants felt were important for inclusion in a research definition of Long COVID in CYP were incorporated into the definition.

### Views of CYP

In order to ensure the voices of CYP were heard, members of the Patient and Public Involvement (PPI) Research Advisory Group (RAG) for the CLoCk Study were invited to attend a virtual meeting to review the statements upon which consensus importance was agreed at the main consensus meeting. The PPI RAG consists of 12 participants who have been recruited to reflect the age range of the CLoCk Study (11–17 years) and to be as representative of the general population as possible, while also including CYP from specific groups including those with long-term health conditions and/or mental health conditions, those from minority ethnic groups and those from lower socioeconomic status areas. Approximately half of the PPI RAG have tested negative and half have tested positive for COVID-19, with some participants experiencing ongoing symptoms. Following review of each statement, participants used the chat function to confirm whether they agreed or disagreed with the statement’s importance in relation to developing a research definition of Long COVID.

## Results

### Participants

One hundred and twenty participants registered to take part in the study. This included 23 people (19%) in the Lived Experience panel, 50 (42%) in the Researcher/Combination panel and 47 (39%) in the Service Delivery panel (table 1). One hundred and five registered participants (88%) completed phase one, 86 eligible participants (82% of those completing phase one) completed phase two and 77 eligible participants (90% of those completing phase two) completed phase three. Seventeen participants attended and voted at the consensus meeting.

**Table 1:** Summary of participants

	Registered for round one	Completing round one	Completing round two	Completing round three	Consensus meeting
Lived Experience	23	22 (96%)	21 (95%)	21 (100%)	2
Researcher/Combination	50	43 (86%)	36 (84%)	31 (86%)	11
Service Delivery	47	40 (85%)	29 (73%)	25 (86%)	4
**Total**	**120**	**105** (**88%**)	**86** (**82%**)	**77** (**90%**)	**17**

### Delphi survey ratings

Following the Delphi process, seven statements were defined as consensus important in two or more panels and were therefore automatically discussed at the consensus meeting. A further three statements were close to two panel ‘consensus important’ and were therefore promoted for discussion at the consensus meeting by the study team. A total of 10 statements were therefore taken forward for discussion at the consensus meeting. Fifteen statements were defined as consensus unimportant in two or more panels and were therefore not eligible for inclusion in the definition. There was one or no panel consensus for the remaining 24 statements, none of which were promoted for discussion or voting at the consensus meeting by the study team or consensus meeting attendees (table 2).

**Table 2:** Delphi phase three—important and less important statements for the definition of post-COVID-19 condition

Statement category	Statement	Important		Less important	
		Three-panel consensus important	Two-panel consensus important	One or no panel consensus important	Three or two-panel consensus unimportant
Testing	At least one positive COVID-19 test		√		
	A positive PCR test for COVID-19			√	
	A positive lateral flow test for COVID-19				√
	An antibody test for COVID-19			√	
Type of initial symptoms	Before or at the time of their COVID-19 test			√	
	During which time they had at least one recorded fever				√
	During which time they lost their sense of smell			√	
	During which time they lost their sense of taste				√
	During which time they had a persistent cough				√
	During which time they had headache				√
	During which time they had unusual tiredness			√	
	During which time they had a sore throat				√
Number of initial symptoms	1 symptom only at the time of testing				√
	2 or more symptoms at the time of testing			√	
	3 or more symptoms at the time of testing				√
	4 or more symptoms at the time of testing				√
	5 or more symptoms at the time of testing				√
Persisting physical symptoms	Persisting unusual tiredness			√	
	Persisting headaches			√	
	Persisting unusual shortness of breath			√	
	Persisting loss of smell or taste			√	
	Persisting dizziness			√	
	1 or more persisting physical symptoms	√			
	2 or more persisting physical symptoms			√	
	3 or more persisting physical symptoms			√	
	4 or more persisting physical symptoms			√	
	5 or more persisting physical symptoms			√	
Persisting well-being symptoms	A young person experiences difficulties with emotions, concentration, behaviour or not being able to get on with other people				√
	A young person has had persistent symptoms of anxiety (worry)				√
	A young person has had persistent symptoms of low mood (sadness)				√
	A young person has had persistent problems with concentration			√	
	The young person’s emotional difficulties have occurred or become worse after COVID-19 infection			√	
Duration	Persist for more than 1 month after initial testing*	√			
	Persist for more than 3 months after initial testing	√			
	Persist for more than 6 months after initial testing			√	
	Persist for more than 1 month after initial testing and are from the list of common symptoms on page 4 (ie, unusual tiredness, headaches, shortness of breath, loss of smell or taste, dizziness)			√	
	Persist for more than 3 months after initial testing and are from the list of common symptoms on page 4 (ie, unusual tiredness, headaches, shortness of breath, loss of smell or taste, dizziness)	√			
	Persist for more than 6 months after initial testing and are from the list of common symptoms on page 4 (ie, unusual tiredness, headaches, shortness of breath, loss of smell or taste, dizziness)			√	
Burden of symptoms	The young person has symptoms that continue or develop after COVID-19 which impact their physical, mental or social well-being	√			
	The young person has symptoms that are interfering with some aspect of daily living (eg, school, work, home, relationships)	√			
	The young person can judge the level of interference with their life themselves			√	
	The level of interference is assessed by a professional			√	
	The impact of the symptoms on functioning is at least moderate†		√		
Tests to exclude other diseases	Persisting COVID-19 antibodies				√
	A negative glandular fever (monospot, antibody or EBV PCR) test†		√		
	A normal full blood count			√	
	An abnormal full blood count				√
	A normal full blood count, CRP, ESR, urea and electrolytes, creatinine, calcium, liver function tests, random blood glucose†		√		
	A normal full blood count, CRP, ESR, urea and electrolytes, creatinine, calcium, liver function tests, random blood glucose, creatine kinase, thyroid function tests, coeliac disease screen, ferritin, vitamin D			√	

Statements closer to three/two-panel consensus important were identified if the percentage of people in each panel rating 7–9 was closer to 70% and 1–3 was closer to <15%.

*Close to three-panel consensus important.

†Close to two-panel consensus important.

CRP, C reactive protein; EBV PCR, Epstein Barr virus polymerase chain reaction; ESR, erythrocyte sedimentation rate.

### Consensus meeting and the views of CYP

Seventeen experts participated in the consensus meeting: 4 (23%) from the Service Delivery panel, 11 (65%) from the Researcher panel and 2 (12%) from the Lived Experience panel. Following discussion and voting in the consensus meeting, 5 of the 10 statements met the threshold for inclusion in the definition of Long COVID in CYP (table 3). Detailed discussion was also held around excluding specific conditions, and there was agreement that it was important that the symptoms experienced by a child or young person needed to be attributable to Long COVID and not to another disease. However, it was also agreed that the definition should not require a particular test for a specific disease to be conducted for the purpose of ensuring that the symptoms were attributable to Long COVID.

**Table 3:** Statements where consensus for inclusion in the definition is achieved or close to consensus and discussed at the consensus meeting

Statement	N (%) voting for inclusion	N (%) of CYP voting for inclusion	Decision
**Testing**			
At least one positive COVID-19 test needed	17 (100)	6 (75)*	Include
**Burden of symptoms**			
The young person has symptoms that continue or develop after COVID-19 which impact their physical, mental or social well-being	17 (100)	8 (100)	Include
The young person has symptoms that are interfering with some aspect of daily living (eg, school, work, home, relationships)	17 (100)	7 (100)*	Include
The impact of the symptoms on functioning is at least moderate	11 (65)	0 (0)	Exclude
**Persisting physical symptoms**			
1 or more persisting physical symptoms	17 (100)	7 (88)*	Include
**Duration**			
Persist for a minimum duration of 12 weeks after initial testing even if symptoms waxed and waned over that period	17 (100)	7 (88)*	Include
Persist for more than 3 months after initial testing and are from the list of common symptoms (ie, unusual tiredness, headaches, shortness of breath, loss of smell or taste, dizziness)	0 (0)	0 (0)	Exclude
Persist for more than 1 month after initial testing	0 (0)	0 (0)	Exclude
**Tests to exclude other diseases**			
A negative glandular fever (monospot, antibody or EBV PCR) test	0 (0)	1 (13)	Exclude
A normal full blood count, CRP, ESR, urea and electrolytes, creatinine, calcium, liver function tests, random blood glucose	0 (0)	0 (0)	Exclude

*One CYP was unable to vote due to technical problems.

CRP, C reactive protein; CYP, children and young people; EBV PCR, Epstein Barr virus polymerase chain reaction.

Eight CYP from the PPI RAG attended a separate virtual session to discuss the Delphi consensus statements. There was broad agreement from the CYP with the statements that had been deemed consensus importance from the main consensus meeting (table 3).

### Included statements for the research definition of Long COVID in CYP

The included statements for a research definition of Long COVID in CYP were as follows:

A condition in which a child or young person has symptoms (at least one of which is a physical symptom) that:

- Have continued or developed after a diagnosis of COVID-19 (confirmed with one or more positive COVID-19 tests).
- Impact their physical, mental or social well-being.
- Are interfering with some aspect of daily living (eg, school, work, home or relationships).
- Persist for a minimum duration of 12 weeks after initial testing for COVID-19 (even if symptoms waxed and waned over that period).

Given the overlap between symptoms of Long COVID in CYP and adults, and the utility of aligning definitions of disease for CYP and adults for continuity, a research definition of Long COVID (post-COVID-19 condition) among CYP based on the Delphi consensus but aligned to the WHO definition^29^ is given in box 3.

Box 3 **Research definition of post-COVID-19 condition (Long COVID) among children and young people aligned to WHO definition**

Post-COVID-19 condition occurs in young people with a history of confirmed SARS-CoV-2 infection, with one or more persisting physical symptoms for a minimum duration of 12 weeks after initial testing that cannot be explained by an alternative diagnosis. The symptoms have an impact on everyday functioning, may continue or develop after COVID-19 infection, and may fluctuate or relapse over time.

## Discussion

Using robust consensus methodology, we derived a research definition for Long COVID in CYP (box 3). This included a total of five statements from the testing, burden of symptoms, persisting physical symptoms and duration statement categories.

To the best of our knowledge, this is the first research definition for Long COVID among CYP. It is comparable with the clinical case definition in adults proposed by the WHO (box 1).^29^ The WHO additionally describes the typical symptoms in adults which are similar to those found in CLoCk.^1^ It is reassuring that the domains (SARS-COV-2 confirmation test, burden of symptoms, persisting symptoms and duration) of this WHO definition overlap with our definition of Long COVID among CYP.

This study has both strengths and limitations. We would argue that the provision of data from the CLoCk Study to inform the process to supplement the literature review was innovative. Although the Delphi consensus process is designed to arrive at a definition in the absence of compelling data, the speed of research in the field meant that by the time the Delphi was in progress, such data were available but not in print and it therefore seemed appropriate to provide that information to participants. The Delphi methodology was robust and modelled on best practice, with the consensus meeting led by an experienced and independent chair. The views of CYP were considered; they voted on the inclusion of statements within the definition; and they were not dominated by adults in a face-to-face panel.

The study also had some limitations. In the final consensus meeting, only two individuals with lived experience were present. However, on no occasion did the participants with lived experience vote differently from the majority of the group. English language was selected and the study was performed primarily within the UK. Given that an aim is to derive a definition to allow international studies to be compared, representation from other countries, including non-English speaking and less developed countries, is desirable. Response rates were typical for studies of this type but there was attrition between rounds.

A final, important point concerns the distinction between a clinical case definition and a research definition of Long COVID. It is understandable that the patient groups representing people with Long COVID are concerned about a definition that could restrict access to services that are needed. In our view, the decision whether a child or young person can see a healthcare professional, access any support needed, or be referred, investigated or treated for Long COVID should be a shared decision involving the young person, their carers and clinicians. The stringent research definition arrived at through this consensus process and the definition aligned to that of the WHO can be used to inform that decision-making process, however, it should not be used as the yardstick by which it is determined if CYP can access care.

## Conclusion

A modified Delphi consensus process has produced a research definition of Long COVID in CYP that complements that proposed by the WHO. They are reassuringly similar. Widespread adoption of this definition would allow comparisons between studies such that a core outcome set can be developed and the prevalence, course and outcome of Long COVID in CYP can be reliably evaluated.

## Data Availability

Data are available upon request.
